# Therapeutic management of intestinal fibrosis induced by radiation therapy: from molecular profiling to new intervention strategies *et vice et versa*

**DOI:** 10.1186/1755-1536-5-S1-S13

**Published:** 2012-06-06

**Authors:** Saad Hamama, Sylvie Delanian, Virginie Monceau, Marie-Catherine Vozenin

**Affiliations:** 1 INSERM U-1030 "Molecular Radiotherapy" Institut Gustave Roussy, Villejuif, France; 2 "Molecular Radiotherapy", Université Paris Sud Paris XI, France; 3 Unité de Radiopathologie, Service Oncologie-Radiothérapie, Hôpital Saint-Louis, APHP, Paris, France

## Abstract

Chronic toxicities of locoregional and systemic oncological treatments commonly develop in long-term cancer survivors. Amongst these toxicities, post-radiotherapeutic complications alter patient's quality of life. Reduction of exposure of normal tissues can be achieved by optimization of radiotherapy. Furthermore, understanding of the fibrogenic mechanisms has provided targets to prevent, mitigate, and reverse late radiation-induced damages. This mini-review shows how (i) global molecular studies using gene profiling can provide tools to develop new intervention strategies and (ii) how successful clinical trials, conducted in particular with combined pentoxifylline-vitamin E, can take benefice of biological and molecular evidences to improve our understanding of fibrogenic mechanisms, enhance the robustness of proposed treatments, and lead ultimately to better treatments for patient's benefice.

## Introduction

Therapeutic management of cancer improved during the past decade and is characterized today by a significant increase in patient's survival rates. Although effective on cure rates, both locoregional and systemic oncological treatments present some concerns related to development of chronic toxicities that alter patient's quality of life, while results of combined therapies suggest that normal tissue toxicity will become a major concern within the next years. Amongst these toxicities, radiation enteropathy is a significant delayed side effect of lumbar and pelvic radiotherapy.

The risk, severity and nature of these radiation-induced toxicities depend on several factors including radiotherapy-related factors (total dose, dose per fraction and volume exposure) and patient-related factors (comorbidities) [1]. Accordingly, a real effort has been made to reduce normal tissue exposure by ballistic and imaging optimization of radiotherapy. Besides technological tools, understanding the fibrogenic mechanisms and targeting profibrotic factors has provided alternate and promising approaches to prevent, mitigate or even reverse late radiation-induced damages [1,2].

If today's clinical practice always aims to limit aggravating factors, current management of radiation-induced damages involves [3]: (i) anti-inflammatory treatments including corticosteroids; (ii) vascular therapy including pentoxifylline (PTX) or hyperbaric oxygen; (iii) antioxidant treatment including superoxide dismutase in the nineties and then combined pentoxifylline-vitamin E (PTX-VitE) in the last decade.

The first part of this mini-review shows how global molecular studies using gene profiling can provide tools to develop new intervention strategies with old molecules or new compounds. The second part shows how successful clinical research done with well-known but low potent old drugs takes benefice of biological and molecular evidences to improve its robustness for patient's benefice.

## From molecular profiling to new intervention strategies

### Cellular and molecular mechanisms involved in the persistence of radiation fibrosis

In all irradiated tissues and especially when vital organs like the heart, lung or intestine are affected [4], the most concerning aspect of radiation fibrosis is its progressive and seemingly irreversible evolution. Thus, the development of curative anti-fibrotic strategies is nowadays highly anticipated by both patients and physicians [5]. Definition of new biologically-based anti-fibrotic strategies is therefore an attractive option to be achieved by characterization of the cellular and molecular mechanisms involved in the persistence of radiation fibrosis.

In human radiation enteropathy, fibrosis is the main histopathological hallmark [6]. Fibrosis contributes to the loss of intestinal compliance and impaired intestinal function and we showed that it was associated with heavy deposition of **Connective Tissue Growth Factor (CTGF/CCN2) **[7]. CTGF gene regulation is known to be under the control of TGF-β *via *a Smad consensus sequence and TGF-β RE/BCE-1 binding sites located in the CTGF promoter region [8,9]. We showed that, surprisingly, **TGF-β1 **expression in fibrotic area was low during the onset of radiation enteropathy [7]. The molecular basis of this paradox was investigated using (i) a high-throughput biological approach by cDNA array profiling and (ii) a classical biochemical approach with recombinant TGF-β1 and CTGF. These studies were performed with unique and physiologically relevant cell models, employing primary smooth muscle cells and sub-epithelial myofibroblasts derived from radiation enteropathy. These cells mimic fibrosis *in vitro*, as they maintain their fibrogenic features in long term culture (6-8 passages) i.e. altered contractile function, modification of the actin cytoskeleton and increased secretory activity [10,11].

The comprehensive cDNA approach showed activation of the **Rho/ROCK pathway **[12]. Further functional *in vitro *experiments showed that this intercellular signaling pathway controls CTGF expression in intestinal smooth muscle cells and in subepithelial myofibroblasts derived from radiation enteropathy [10,13]. In addition, our gene profiling studies showed that radiation enteropathy was associated with a global deregulation of the **extracellular matrix **remodeling with increased ECM deposition, MMPs and TIMPs activity [14]. Whether this dynamic remodeling process was the cause or the consequence of the phenotypic alteration of the resident mesenchymal cells is currently under investigation.

Cell response to increasing concentrations of recombinant TGF-β1 was investigated. Activation of cell-specific signaling pathways by low TGF-β1 concentrations was demonstrated with a prominent activation of the Rho/ROCK pathway in fibrosis-derived cells, whereas the Smad pathway was predominantly activated in normal cells. This differential fibrogenic response identified in normal *versus *fibrosis-derived cells opened new therapeutic opportunities for targeted anti-fibrotic therapy. In addition, we showed that recombinant CTGF was able to trigger its auto-induction in fibrosis-derived cells, an effect which was further enhanced by TGF-β1. These results thus identify specific and combinatorial roles of low TGF-β1 doses and CTGF for the maintenance of tissue fibrosis [15].

### From physiopathological mechanisms to clinical transfer

**Figure 1:**
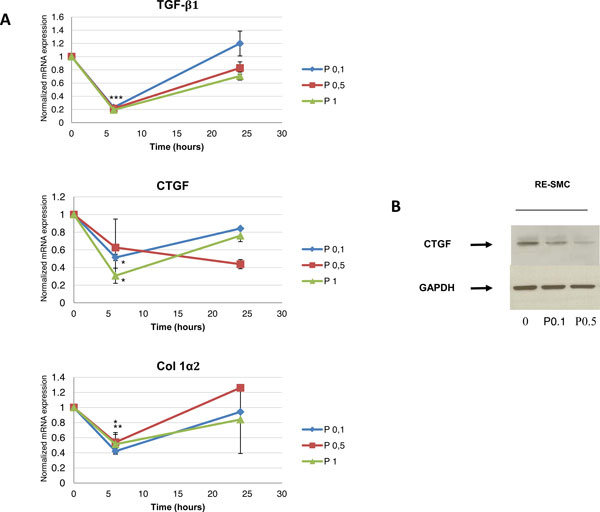
**A. Effect of Pravastatin on mRNA expression of TGFβ1, CTGF and Collagen in a kinetic manner**: Twenty four hours kinetics of mRNA expression of TGFβ1, CTGF, and Col Iα2 in control and Pravastatin treated cells show that Pravastatin treatment with a dose of 0.1 mM and up reduces levels of mRNAs of these genes with maximum efficiency at six hours post-treatment; C: control, P: Pravastatin treatment. 0.1, 0.5, and 1 refer to treatment dose in mM. *: p < 0.05, **: p < 0.01, ***: p < 0.005 according to kruskal-Wallis test. **B.Protein expression of CTGF in control and Pravastatin treated cells: **Pravastatin treatment for 24 hours inhibits CTGF protein expression showing also a dose-response relationship. GAPDH is used as a housekeeping gene. C: control, P: Pravastatin treatment. 0.1, 0.5 refer to treatment dose in mM.

Rho GTPases regulate fundamental cellular processes including cell motility, cell cycle progression, cell survival, transcription, membrane trafficking and cytokinesis *via *their downstream effectors the Rho-associated kinases (ROCKs) [16,17]. Many Rho functions have been elucidated using pharmacological inhibitors, the most prominent ones being Statins, molecules which inhibit isoprenoid intermediates production and Rho activation. In order to investigate whether the Rho/ROCK cascade regulates radiation-induced fibrogenic program in intestinal mesenchymal cells, pharmacological inhibition of Rho and ROCK activation was performed *in vitro *using pravastatin and Y-27632, a pyrimidine derivative inhibitor of ROCK. We showed that both agents modulated radiation-induced fibrogenic differentiation and the expression of CTGF, TGF-β1, and collagen Iα2 genes (Figure 1), most likely *via *NF-κB inhibition [10,11,18]. Next, therapeutic experiments were conducted in pre-clinical models. Pravastatin was chosen as, in the case of convincing results, it would be easy to accelerate the transfer of this drug to the clinic, given the fact that the drug is safe and well tolerated [19]. Remarkably, we showed that pravastatin administration with curative intent improves radiation enteropathy in rats, inhibits Rho and ROCK activity in human samples, and subsequently inhibits CTGF production *in-vivo, ex-vivo*, and *in-vitro*. In addition, inhibition of type-I-collagen and fibronectin occurred, indicating that pravastatin modulates the secretory phenotype of mesenchymal cells, probably by inhibition of the Rho/ROCK/CTGF/ECM cascade [13]. Mitigation experiments with pravastatin, relevant to clinically well-established fibrosis, improved delayed radiation enteropathy in rats and decreased both CTGF expression and collagen deposition. Interestingly, pravastatin's protective effect was differential, as no tumor protection occurred [20]. Similar results were obtained by others using Simvastatin [21] and anti-fibrotic efficacy of statins was shown in model of radiation-induced lung [22,23]. These pre-clinical findings encouraged us to propose a phase II randomized clinical trial, which received approval from the local ethics committee and started at Institut Gustave Roussy in January 2010 with the support of the French Ministry of Health (PHRC 2010).

Searching for anti-fibrotic treatment using molecular targets lead to the development of targeted drugs. Amongst them anti-CTGF antibody [24], imatinib (anti-PDGF antibody) [25-27], statin (Rho/Rock-inhibitors) [20-23,28] have demonstrated efficacy in pre-clinical studies and need now to be validated in clinical trials. Two targeted drugs have recently showed interesting results in recent trials: the small molecule inhibitor of TGF-β1, pirfenidone, and the tyrosine kinase inhibitor, BIBF 1120. Both of them halt progression of idiopathic pulmonary fibrosis [29,30]. Still others, such as combined pentoxifylline-vitamin E has a proven clinical efficacy with an unknown mechanism of action [31-38].

## From clinical trials to understanding the molecular mechanism

In the nineties an antioxidant compound, the liposomal Cu/Zn superoxide dismutase (SOD), was the first drug to half reduce established radiation fibrosis in patients and experimental models (reviewed in [3]). Biological studies showed that SOD inhibited TGF-β1 and TIMPs and lead to a subsequent reversion of the myofibroblastic phenotype in vitro [39,40]. Later, Delanian *et al*. showed that combined PTX-Vit.E administered to patients, half reduced superficial radiation fibrosis in 6 months [31]. Results were confirmed in a randomized clinical trial [32] and by long-term clinical results (3 years) [33]. A first experimental study using skin fibrosis in pig as model showed that the histopathological recovery after PTX/VitE treatment was associated with significant reduction of TGF-β1 deposition [41]. Since then, numerous clinical trials were successful and showed improvement of osteoradionecrosis [34], superficial fibrosis [36], proctitis/enteritis [37], pelvic complications [38], uterine fibro-atrophy [35,42]. Moreover, combined PTX-Vit.E in rat experiment had beneficial effects on radiation-induced myocardial fibrosis and left ventricular function [43]. Recent report using a triple combination PENTOCLO: PTX-VitE and clodronate; a biphosphonate active against macrophages also allows successful healing of severe osteoradionecrosis [44] and reduction of neurological symptoms associated with radiation-induced plexopathies [45]. The efficacy of this triple combination is currently validated in a phase III randomized clinical trial that has started at Saint Louis Hospital for radiation-induced plexitis (support by the French Ministry of Health, PHRC 2009). Therefore, understanding the molecular mechanism of PTX-Vit.E combination solicit much interest for better understanding of radiation-induced fibrosis, to improve treatment robustness and will pave the way toward new more efficient treatments.

Vitamin E (α-tocopherol) is an antioxidant drug known to modulate the expression of several genes such as ICAM-1 integrins and PPAR-gamma [46]. α-tocopherol was reported to decrease the expression of genes known to be involved in the fibrotic process such as (MMP-1) in human skin fibroblast [47], and IL-1β in THP-1 cells [48]. In mice, D-α-tocopherol supplementation decreased collagen mRNA in the liver by 70% [49]. In type 2 diabetic patients, α-tocopherol supplementation lowered plasma levels of PAI-1 and P-selectin [50]. In other case, α-tocopherol induces the expression of CTGF in human smooth muscle cells while neither β-tocopherol nor N-acetylcysteine do [51]. It was suggested that this modulation of CTGF was unique as it was not triggered by structurally related antioxidant molecules, suggesting occurrence of a non-antioxidant mechanism in the transcriptional regulation of several genes.

Pentoxifylline, PTX is a methylxanthine derivative used to treat vascular disease such as intermittent claudication. *In vivo*, it has been reported to have anti-TNF-α effect, increase erythrocyte flexibility, vasodilate, and inhibit inflammatory reactions. *In vitro *studies have indicated that PTX has also antioxidant properties [52], inhibits human dermal fibroblast proliferation and extracellular matrix production [53-55] and increases collagenase activity [53]. However, the doses of pentoxifylline required to produce these effects *in vitro *are high, and reached 1000 μg/ml in some cases [54] rendering *in vivo *use of PTX unsuitable. In addition, PTX is known as a non-specific phosphodiesterases inhibitor that subsequently increases intracellular levels of cAMP. Like other cAMP elevating agents PTX could activate protein kinase A (PKA) which would phosphorylate transcription factors, such as cAMP response element binding protein (CREB). Activated CREB recruits the coactivators CBP and P300 that also act as transcriptional co-activators for SMADs [56,57]. Therefore, the sequestration of CBP/P300 by activated CREB could inhibit SMAD-dependent transcription [58] and constitute one molecular mechanism to explain PTX anti-fibrotic action that remains to be demonstrated *in vivo*. For example, in a model RIF-induced in pigs no clinical or histological changes were observed in RIF after 6 months of treatment with PTX alone using maximum tolerated dose [41]. Extrapolation from *in vitro *studies would however suggest that higher concentration of PTX would be required to achieve effective suppression of collagen synthesis or to increase collagenase activity. Used at this dosage, PTX might be extremely toxic and suggests that administration of PTX alone does not constitute an anti-fibrotic treatment.

**Figure 2:**
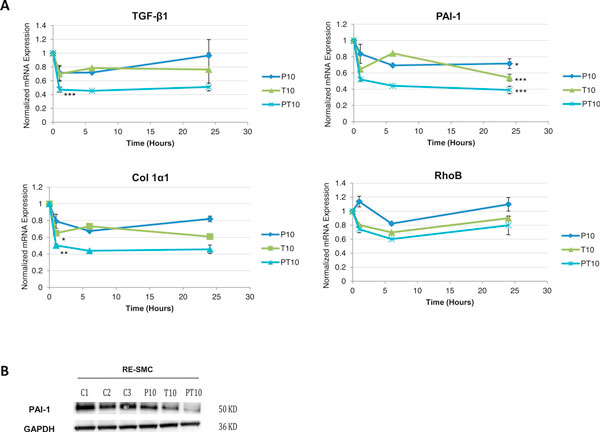
**A. Effect of combined pentoxifylline-trolox on mRNA expression of profibrotic genes in a kinetic manner**: Twenty four hours kinetics of mRNA expression of TGF-β1, PAI-1, Col Iα1, and RhoB. Pentoxifylline and trolox synergize to inhibit TGFβ1 at early point time, subsequent inhibition of TGFβ1 targets such as PAI-1 and Col Iα1. Treatment doesn't affect RhoB mRNA expression. P: Pentoxifylline treatment, T: Trolox treatment, PT: Pentoxifylline-trolox treatment. 10 refers to treatment dose in μg/ml. *: p < 0.05, **: p < 0.01, ***: p < 0.005 according to kruskal-Wallis test. **B.Protein expression of PAI-1 in control and pentoxifylline-trolox treated cells**: Combined pentoxifylline-trolox treatment for 24 hours inhibits PAI-1 protein expression more effectively than pentoxifylline alone or trolox alone; GAPDH is used as a housekeeping gene. C: control, P: Pentoxifylline treatment, T: Trolox treatment, PT: Pentoxifylline-trolox treatment. 10 refers to treatment dose in μg/ml.

Studies about the mechanism of action of combined pentoxifylline-vitamin E in radiation fibrosis are awfully limited. An *in vitro *study was conducted in dermal fibroblast using the water-soluble analogue of vitamin E, trolox, to investigate the effects of combined pentoxifylline-trolox on irradiated cells. This study showed reduction in acute and late ROS formation in cells after irradiation, decrease in DNA strand breaks whenever the drugs were added i.e. before or after irradiation supporting an immediate anti-oxidant action that interfere with the DNA repair process [59]. However, the relevance of this study to fibrosis is unclear since only short term response was investigated, therefore we aimed at investigating the role of combined pentoxifylline-vitamin E on two well know fibrogenic pathway, i.e. TGF-β1 and Rho/Rock using an *in vitro *model of radiation-induced fibrosis consisting of primary smooth muscle cells derived from human radiation enteropathy samples (RE-SMC). The hydrophilic analogous of α-tocopherol, trolox, was used. Incubation of the cells with combined pentoxifylline-trolox didn't regulate RhoB mRNA expression (Figure 2A) nor influence Actin cytoskeleton in RE-SMC (data not shown) but interestingly negatively modulated TGF-β1 mRNA expression at early time point (one hour post-treatment) and subsequently decrease the expression of TGF-β1 targets such as PAI-1 both at mRNA and protein levels (twenty four hours post-treatment) (Figure 2A and 2B). This suggested that the anti-fibrotic effects of combined pentoxifylline-trolox could be mediated by **inhibition of the TGF-β1 pathway**. Interestingly, pentoxifylline and trolox appear to enhance the activity of each other; and the effect of the combination was more potent than any of the individual treatments, which is the definition of drugs **synergy**. Combined pentoxifylline-trolox with a dose of 10 μg/ml decreases protein expression of PAI-1 more effectively than trolox alone (10 μg/ml) or pentoxifylline alone (10 μg/ml) (Figure 2B). Thus, the synergy between the elements of this combination at low concentration could constitute the basis of its efficacy conferring a more appropriate therapeutic window. This study offers for the first time, to our knowledge, a molecular base for rationalization of the clinical use of combined Pentoxifylline-vitamin E in radiation fibrosis. Nevertheless, inhibition of TGF-β1 pathway is unlikely to be the sole anti-fibrotic mechanism of action of combined pentoxifylline-trolox and other novel candidates are actually under investigations.

## Conclusion

From molecular profiling to clinical trials in a bottom-up manner or from the clinical trials to the molecular understanding in a top-down approach, these studies have optimized our understanding of radiation-induced fibrogenesis. Combining these information could ultimately lead to improve management of fibrosis.

In a model of intestinal fibrosis, Statins act principally via inhibition of Rho/Rock pathway decreasing subsequently the expression of CTGF. In contrast, combined pentoxifylline-trolox inhibits TGF-β1 pathway while it appears to have no influence on Rho/Rock pathway. Therefore combining these two medications to generate a more efficient triple therapy seemed a logical follow up. While emerging new combinations might be promising, heavy investigations are needed to prove their safety and efficacy over already solid candidates as Statins or combined pentoxifylline-vitamin E.

## Competing interests

The authors declare that they have no competing interests.
